# Prevalence and diversity of *Salmonella enterica* in water, fish and lettuce in Ouagadougou, Burkina Faso

**DOI:** 10.1186/s12866-015-0484-7

**Published:** 2015-07-31

**Authors:** Oumar Traoré, Outi Nyholm, Anja Siitonen, Isidore Juste O Bonkoungou, Alfred S Traoré, Nicolas Barro, Kaisa Haukka

**Affiliations:** Laboratoire de Biologie Moléculaire d’Epidémiologie et de Surveillance des bactéries et virus transmis par les aliments, CRSBAN/Département de Biochimie-Microbiologie, UFR SVT-Université de Ouagadougou, 03 B.P. 7021, Ouagadougou, 03 Burkina Faso; Laboratoire National de Santé Publique, 09 B.P. 24, Ouagadougou, 09 Burkina Faso; Bacteriology Unit, Department of Infectious Disease Surveillance and Control, National Institute for Health and Welfare (THL), P.O. Box 30, FI-00271 Helsinki, Finland; Department of Food and Environmental Sciences, University of Helsinki, P.O. Box 56, FI-00014 Helsinki, Finland

**Keywords:** *Salmonella*, Serotypes, Water, Fish, Lettuce, Antimicrobials

## Abstract

**Background:**

This study investigated the prevalence, serotypes and antimicrobial sensitivity patterns of *Salmonella enterica* in environment in Ouagadougou, Burkina Faso. A total of 476 samples, consisting of 36 samples of tap water, 51 samples of well water, 87 samples of channel water, 44 samples of reservoir water, 238 samples of fish, and 20 samples of lettuce were examined using standard bacteriological procedures for *Salmonella*.

**Results:**

*Salmonella* were isolated from 98 samples. *Salmonella* were rare in drinking water, since they were not found at all from the tap water, and only in 2 % of well water. *Salmonella* were more common in the water of reservoir of Tanghin (15 %), reservoir of Yamtenga (20 %), and in the water channels in the city (from 20 to 31 %). *Salmonella* were commonly isolated from the fish (24 %) caught from the reservoir of Tanghin and from the lettuce (50 %) irrigated with water from Tanghin. The *Salmonella* isolates were found to represent 50 different serotypes. The 11 most common serotypes were *Salmonella* Bredeney and *S*. Colindale (both 8.2 %), *S*. Muenster (6.1 %), *S.* Korlebu (5.1 %), *S.* Eastbourne and *S.* Poona (both 4.1 %), and *S.* Agona, *S.* Derby, *S.* Drac, *S.* Senftenberg, *S.* Waycross (each 3.1 %), accounting for 51.3 % of all the isolates. In general, the *Salmonella* strains were sensitive to the antimicrobials tested, but two strains were resistant to streptomycin and many more intermediate to streptomycin or sulphonamide.

**Conclusion:**

This study highlights the common prevalence of *Salmonella* and the high diversity of *Salmonella* serotypes in aquatic environment in Ouagadougou, Burkina Faso. Therefore, various human activities linked to water and consumption of water-related products, such as fish and lettuce, can lead to human *Salmonella* infections.

## Background

Microbiologically contaminated water is a potential source of human enteric infections and indicates poor maintenance of hygiene-related infrastructure and problems in implementation of control measures especially in developing countries*.* Discharge of inadequately treated sewage, run-off storm water and leakage of animal waste into the environment can lead to deterioration of quality of water sources. *Salmonella* has been found to survive in tropical fresh waters, such as rivers, streams and wells in Sierra Leone, for several days [1]. *Salmonella* carried by fish and other aquaculture products as well as by fresh produce irrigated with dirty water has been indicated as a vehicle for a growing number of enteric disease outbreaks [2–6]. Use of feces-contaminated water to irrigate the produce has been reported to lead contaminating of soil and vegetables with *Salmonella* for an extended period of time [7, 8].

*Salmonella enterica* remains an important cause of diarrhoeal illness in humans and *Salmonella* infections are a major health concern that continue to have a serious economic impact worldwide [9, 10]. Furthermore, specifically in sub-Saharan Africa, non-typhoidal *Salmonella* is among the leading causes of bacterial bloodstream infections in adults and children [11–13]. Yet, the common sources of *Salmonella* infections in Africa are poorly known.

In our previous study in Ouagadougou in Burkina Faso, 9 % of the children suffering from diarrhea were found to be infected by *Salmonella* [14]. In order to find out the potentially important sources for the *Salmonella* infections, we examined the frequency and diversity of *Salmonella* in beef, mutton and poultry carcasses [15, 16] as well as in the feces of cattle, poultry, swine and hedgehogs [17]. In the present study we expand our studies on the potential *Salmonella* sources to include water, fish and irrigated lettuce.

In Ouagadougou, channels built to drain runoff rain water are used as a human biological waste deposit by people living close to them. Even a major hospital disposes much of its waste into a channel running nearby, casting serious doubt on the hygienic quality of the water. Elsewhere in Africa similar open storm drainage channels have been found to expose people to infections by *Salmonella* and other pathogens [18]. The channels in Ouagadougou run into reservoirs, which are nowadays used for fishing activities, irrigation of vegetables, washing clothes and cars. Furthermore, the reservoirs remain a contingency source of drinking water during dry season being intermittently part of municipal infrastructure. In this study, we examined water samples from two reservoirs, two channels as well as from wells and treated tap water system for possible *Salmonella* contamination. Furthermore, we examined fish caught from one of the reservoirs and lettuce irrigated with its water. Specifically, the aims of this study were (1) to determine the prevalence of *Salmonella*, (2) to assess *Salmonella* serotypes and antimicrobial resistance profiles of the obtained isolates and (3) to compare the serotypes to those previously obtained from the local children, animals and retail meat.

## Results

*Salmonella enterica* was isolated from 98 (20,6 %) of the total of 476 samples examined (Table 1). From all of the drinking water samples (taps and wells) only one isolate was obtained. Instead, in the channel, reservoir and fish samples *Salmonella* was common, 15-31 % of the samples were positive (Table 1). In the lettuce samples the frequency was the highest, since half of them were positive.

**Table 1:** Prevalence of *Salmonella* and different serotypes detected

Sample types		Examined	Positive (%)	Number of serotypes
Tap water		36	0	0
Well water		51	1 (2 %)	1
Channel water	CA	52	16 (31 %)	13
	CB	35	7 (20 %)	7
Reservoir water	RY	10	2(20 %)	2
	RT	34	5 (15 %)	4
Fish		238	57 (24 %)	34
Salad		20	10 (50 %)	4
Total		476	98 (21 %)	50

CA = Channel A, CB = Channel B, RY = Reservoir of Yamtenga RT = Reservoir of Tanghin

The obtained 98 *Salmonella enterica* isolates represented 50 different serotypes, all the sample types containing a variety of serotypes (Table 1). The most frequent serotypes were *Salmonella* Colindale and *S.* Bredeney (8 isolates each) (Table 2). *Salmonella* Colindale was isolated from lettuce as well as from the reservoirs and channels, whereas another common serotype in lettuce, *S.* Korlebu, was not detected in the water samples. *S*. Bredeney was common in fish but was also detected in water and lettuce samples (Table 2). *S*. Bredeney, *S.* Give, *S*. Colindale, *S.* Eastbourne, *S*. Schwarzengrund, *S*. Poona and *S.* Llandoff were isolated from fish and water samples, but only *S.* Colindale and *S.* Schwarzengrund were isolated from both fish and the reservoir of Tanghin where they were caught from. No common serotypes were found from the two different reservoirs. Only two serotypes, *S.* Colindale and *S.* Senftenberg were common to the two different channels.

**Table 2:** Diversity of *Salmonella* serotypes

Serotypes	Origins								Total (*n* = 476)
	Water						Fish (*n* = 238)	Lettuce (*n* = 20)	
	Tap (*n* = 36)	Well (*n* = 51)	Channel (*n* = 87)		Reservoirs (*n* = 44)				
			CA (*n* = 52)	CB (*n* = 35)	RY (*n* = 10)	RT (*n* = 34)			
*S*. Adabraka	0	0	0	0	1	0	0	0	1 (1 %)
*S*. Agona	0	0	0	0	0	0	3	0	3 (3 %)
*S*.Bredeney	0	0	1	0	0	0	6	1	8 (8.2 %)
*S*. Brive	0	0	0	0	0	0	1	0	1 (1 %)
*S*. Brochum	0	0	0	0	0	0	2	0	2 (2 %)
*S*. Carmel	0	0	0	0	0	1	0	0	1 (1 %)
*S*. Chester	0	0	1	0	0	0	0	0	1 (1 %)
*S.* Colindale	0	0	1	1	0	2	0	4	8 (8.2 %)
*S*. Cubana	0	0	0	0	0	0	1	0	1 (1 %)
*S*. Derby	0	0	0	0	0	0	3	0	3(3.1 %)
*S*.Drac	0	0	0	0	0	0	3	0	3 (3.1 %)
*S*. Ealing	0	0	0	0	0	0	1	0	1 (1 %)
*S*. Eastbourne	0	0	3	0	0	0	1	0	4 (4.1 %)
*S*. Eastglam	0	0	0	0	0	0	2	0	2 (2 %)
*S.* Elisabethwille	0	0	0	0	0	0	1	0	1 (1 %)
*S*. Freshno	0	0	0	0	0	0	1	0	1 (1 %)
*S*. Galiema	0	0	0	1	0	0	0	0	1 (1 %)
*S*. Gerland	0	0	0	0	0	0	0	1	1 (1 %)
*S*. Give	0	0	0	1	0	0	1	0	2 (2 %)
*S*. Havana	0	0	0	0	0	0	2	0	2 (2 %)
*S.* Hermannsweder	0	0	0	0	0	0	1	0	1 (1 %)
*S*. Kentucky	0	0	0	0	0	0	1	0	1 (1 %)
*S*. Kokomlemle	0	0	0	0	0	0	1	0	1 (1 %)
*S*.Korlebu	0	0	0	0	0	0	1	4	5 (5.1 %)
*S*. Llandoff	0	0	1	0	0	0	1	0	2 (2 %)
*S*. Mentevideo	0	0	0	0	0	0	1	0	1 (1 %)
*S.* Mbandaka	0	0	0	0	0	0	1	0	1 (1 %)
*S.*Minnesota	0	0	0	0	0	0	1	0	1 (1 %)
*S.* Muenster	0	0	0	0	0	0	6	0	6 (6.1 %)
*S.* Nima	0	0	0	0	0	0	1	0	1 (1 %)
*S.* Nottingham	0	0	0	0	0	0	2	0	2 (2 %)
*S*. Offa	0	0	0	0	0	0	1	0	1 (1 %)
*S*.Ouagadougou	0	0	1	0	0	0	0	0	1 (1 %)
*S*. Poona	0	1	0	1	0	0	2	0	4 (4.1 %)
*S*. Rissen	0	0	0	1	0	0	0	0	1 (1 %)
*S*. Schwazengrund	0	0	0	0	0	1	1	0	2 (2 %)
*S*.Senftenberg	0	0	2	1	0	0	0	0	3 (3.1 %)
*S*. Shubra	0	0	1	0	0	0	0	0	1 (1 %)
*S*. Teshie	0	0	1	0	0	0	0	0	1 (1 %)
*S*. Tilene	0	0	1	0	0	0	1	0	1 (1 %)
*S*. Typhimirium	0	0	0	0	0	0	1	0	1 (1 %)
*S*. Virchow	0	0	1	0	0	0	0	0	1 (1 %)
*S*. Waedenswil	0	0	0	0	0	0	1	0	1 (1 %)
*S*. Wagadugu	0	0	0	1	1	0	0	0	2 (2 %)
*S*. Waycross	0	0	0	0	0	0	3	0	3 (3.1 %)
*S*. Group A	0	0	0	0	0	0	1	0	1 (1 %)
*S*.GroupB:4,12,27:z35:l,w	0	0	0	0	0	0	1	0	1 (1 %)
*S*.GroupE1,3,19;r:-	0	0	1	0	0	0	0	0	1 (1 %)
*S*.GroupG13,22:z:-	0	0	0	0	0	1	0	0	1 (1 %)
*S*. Group 0:53	0	0	1	0	0	0	0	0	1 (1 %)
Total	0	1	16	7	2	5	57	10	98

CA = Channel A, CB = Channel B, RY = Reservoir of Yamtenga RT = Reservoir of Tanghin

Almost all of the isolates were sensitive to the 12 antimicrobials tested. Only two isolates, *S.* Wagadugu and *S.* Adabraka isolated from the water of the reservoir of Yamtenga, were resistant to streptomycin. However, 27 of all the 98 isolates showed decreased sensitivity (i.e. were intermediate) to streptomycin and 5 to sulphonamide.

## Discussion

Our investigation on prevalence of *Salmonella enterica* in water from taps, wells, channels and reservoirs, fish, and lettuce grown in Ouagadougou, Burkina Faso, indicated that *Salmonella* contamination is rare in drinking water but common in the samples from the other sources (15-50 % prevalence). In a previous study in Lagos, Nigeria, *Salmonella* were isolated from 18,5 % of drinking water samples [19]. Serotyping of the *Salmonella* isolated in our study revealed the great diversity of serotypes in these sources: we identified 50 different serotypes among the 98 isolates. Recently, in Burkina Faso, 383 *Salmonella* strains were isolated from animal faeces (cattle, poultry, swine and hedgehogs), representing 81 different serotypes [17]. Of those, Agona, Bredeney, Brive, Carmel, Chester, Colindale, Derby, Drac, Eastbourne, Fresno, Galiema, Kokomlemle, Korlebu, Muenster, Nima, Nottingham, Poona, Rissen, Schwarzenground, Senftenberg, Typhimurium and Virchow were isolated also in our study indicating a possible transfer between animals, their faeces, water, fish and irrigated vegetables. The previously most commonly reported serotypes in Africa (Cameroon, Mali, Marocco, Senegal and Tunisia), Enteritidis and Anatum [20], were not isolated in our study. Serotypes Muenster (the second most common serotype isolated from fish), Cubana, Kentucky, Montevideo, Poona, Fresno, Virchow and Typhimurium detected in our study, were also isolated in stool specimens from children under 5 years old suffering from acute diarrhea in Ouagadougou and in rural Burkina Faso [14, 21].

*Salmonella* were isolated from 23 % of the surface water samples (channels and reservoirs). This frequency is lower than in the survey conducted in Yaoundé, Cameroon, where *Salmonella* were isolated in 49.4 % of the surface water samples [22]. We isolated 22 different serotypes from surface water, showing more diversity in *Salmonella* than reported elsewhere [23–26]. No same serotypes were found from the two reservoirs studied, which may be due to their different external factors. The reservoir of Tanghin located in the city is influenced by people and the reservoir of Yamtenga in the rural area is less frequented by people but more by animals. In any case, the presence of *Salmonella* in the surface waters reveals the health risk posed by the use of this water for irrigation. It has been indicated that the potential hazards may be associated with poor hygienic standard of waters influenced by human sewage, livestock farming, or industry [27]. *S.* Poona, the only *Salmonella* serotype we isolated from well water, was also isolated from children under 5 years suffering from acute diarrhoea in Ouagadougou and in rural Burkina Faso [14, 21].

Among the fish samples examined, 57 (24 %) were positive to *Salmonella* with high diversity of serotypes, 34 different ones were detected. In Kenya, along the Lake Victoria Beaches, almost 17 % of Tilapia samples were positive for *Salmonella* with four different serotypes [28]. In Indonesia, about 10 % of fresh fish were reported to be contaminated by *Salmonella* [5]. The high prevalence of *Salmonella* in fish in Ouagadougou is not surprising since they were caught from water of poor hygienic quality. *S*. Schwarzengrund and Colindale were the serotypes found in both the fish and the reservoir of Tanghin. Although as many as 34 different *Salmonella* serotypes were detected in the fish from Tanghin, only four serotypes were detected in its water, presumably because the salmonellas are more sparsely distributed in the water environment and thus in water samples. *S*. Typhimurium, an important clinical serotype, was isolated only from one fish sample (0,4 %) in our study, thus, being much rarer than in the study of fish along the Lake Victoria Beaches, where it was more isolated (8 %) [28]. In another study, conducted in Winam Gulf of Lake Victoria, Kenya, *S*. Typhimurium was isolated from 6 % of fish harvested [29]. We did not find any *S*. Typhi, perhaps because the enrichment technique used is not optimal to this serotype [30].

Our results revealed that 50 % of the analysed lettuce samples were positive for *Salmonella* with four different serotypes (*S.* Bredeney, *S.* Colindale, *S.* Gerland and *S*. Korlebu).

*S.* Colindale was found in lettuce, reservoir water of Tanghin and the channels. This suggested that the lettuce has been contaminated by irrigating water. On the other hand, *S*. Korlebu and *S*. Gerland found in lettuce samples were not detected in the water sources, but *S*. Korlebu has instead been identified in cattle faeces in Ouagadougou [17].Bacteria in lettuce might also come from another origin e.g. manure used as a fertilizer. *Salmonella* has been demonstrated to contaminate vegetables (carrots, radish, lettuce and parsley) in field experiments following treatment with contaminated manure compost or irrigation water [7, 31]. In Finland, iceberg lettuce contaminated with *S.* Reading and Newport caused a nationwide outbreak*,* according to a traceback investigation [32]. *S.* Colindale which was common in our lettuce samples was also described as the most common serotype in Gambia of non-typhoidal *Salmonella* in cases of enteric infection under the age of 5 years [10].

Reassuringly, our results showed that the great majority of environmental *Salmonella* strains are susceptible to the antimicrobials tested. Two strains, *S.* Wagadugu and *S*. Adabraka, both isolated from the reservoir water of Yamtenga were resistant to streptomycin. Domestic animals treated by antibiotics might transfer resistant *Salmonella* from their faeces to this reservoir leading to antimicrobial resistance. No resistant *Salmonella* were detected in fish reflecting the fact that antimicrobial agents are not used in fish farming in Burkina Faso. On the contrary, 52 (14 %) of the *Salmonella* isolates from animal faeces in Ouagadougou were resistant to one or more tested antimicrobials [17]. In Nigeria, emerging multidrug-resistant *Salmonella* serotypes were commonly found from water sources [19].

## Conclusion

Our study highlights the common presence of *Salmonella* in samples from different aquatic environments and indicates potential sources of human *Salmonella* infections. We detected a high diversity of serotypes among the strains, which were, however, mainly fully sensitive for the common antimicrobials. But nearly 30 % of the isolates were intermediate, which gives rise to concern. These results indicate a need for microbiological quality monitoring of the fish production and water, which is used in various human activities eg. in irrigation of fresh vegetables.

### Methods

#### Sampling

The first sampling period was from October 2008 to February 2009 (corresponding to the dry season) in different parts of Ouagadougou and Yamtenga out of the town. The samples included 36 water samples from 4 taps (9 samples from each tap), 51 samples from 3 wells (17 samples from each well), 11 samples from a channel (Channel A), water of which flows directly to the reservoir of Tanghin, 21 samples from a channel, which runs past a hospital (Channel B) and close to the reservoir of Tanghin (during the rainy season the waters of Channel B and the reservoir overflow to each other), 9 samples from the reservoir of Tanghin and 10 samples from the reservoir of Yamtenga, which is not influenced by channel water. Water from wells and taps were collected as the users did, by lifting with a bucket from the well or taking directly from the tap. Water samples from the reservoir and the channel were taken with a disinfected ladle fixed to a long stick [33]. The samples were transported to the laboratory on ice and analyzed within two hours after collection.

The second sampling period was from March to August 2010 in Ouagadougou. The period from March to May is part of dry season, and the period from June to August part of rainy season. The samples included 25 water samples from the reservoir of Tanghin, 41 from Channel A, 14 from Channel B and 238 fish (tilapia, *Oreochromis niloticus*) from the reservoir of Tanghin. The fish were an average length of 12 cm, purchased alive from local fishermen and processed in the laboratory within four hours after collection. The outer surface of the killed fish was disinfected by wiping with 70 % ethyl alcohol for two minutes, followed by three washings with sterile water. The intestine was removed aseptically by using a scalpel and ground with a sterile mortar and pestle [33]. Lettuce grown in one garden using irrigation water from the reservoir of Tanghin was also sampled. Ten samples consisting of inner leaves of lettuce were placed in sterile plastic bags, kept in an icebox and processed in a laboratory the same day.

#### Microbiological analyses

Broth enrichment technique was used to enhance detection of *Salmonella* strains 1 g of each crushed fish intestine was added to 9 ml of buffered peptone water (BPW, Liofilchem, Italy) and 25 g of lettuce was weighed and added to 225 ml of BPW, stomached and incubated at 37 °C for 18–20 h. From drinking water samples (taps and wells), one litre was filtered through the membrane with 0,45 μm pore size and the membrane was transferred into 90 ml of buffered peptone water (BPW). Reservoir and channel waters were so turbid that 10 ml of water sample was directly added to 90 ml of BPW and incubated at 37 °C overnight. Next day, 0.1 ml from each BPW broth was added to 10 ml of Rappaport-Vassiliadis broth (Oxoid, England) and incubated at 42 °C for 24 h. A loop-full of enriched broth was streaked onto the XLD agar (Oxoid, Basingstoke, England) and incubated at 37 °C for 24 h. Identity of the colonies with black centre was confirmed biochemically using urea, indole, ONPG, Kligler Hajna (Liofilchem, Italy), lysine decarboxylase, citrate and mannitol tests. API 20E strips (BioMérieux, Marcy l’Etoile, France) were used for further confirmation.

The isolates were conserved in heart infusion broth with 15 % of glycerol in the freezer and sent to the Bacteriology Unit the National Institute for Health and Welfare (THL) in Finland for serotyping according to the Kaufmann-White scheme [34]. All isolates were also tested for susceptibility to 12 different antimicrobial agents using the disk diffusion method on Mueller Hinton II agar (Oxoid, England) following the EUCAST guidelines (http://www.eucast.org). *E. coli* ATCC 25922 was used as a control. The antimicrobial disks (Oxoid) used were nalidixic acid (30 μg), ciprofloxacin (5 μg), ampicillin (10 μg), cefotaxime (5 μg), imipenem (10 μg), tetracycline (30 μg), gentamicin (10 μg), chloramphenicol (30 μg), streptomycin (10 μg), sulphonamide (300 μg), mecillinam (10 μg) and trimethoprim (5 μg).

## References

[1] Wright R (1989). The survival patterns of selected faecal bacteria in tropical fresh waters. Epidemiol Infect.

[2] Lynch MF, Tauxe RV, Hedberg CW (2009). The growing burden of foodborne outbreaks due to contaminated fresh produce: risks and opportunities. Epidemiol Infect.

[3] Amagliani G, Brandi G, Schiavano GF (2012). Incidence and role of *Salmonella* in seafood safety. Food Res Int.

[4] Jacobsen CS, Bech TB (2012). Soil survival of *Salmonella* and transfer to freshwater and fresh produce. Food Res Int.

[5] Kusumaningrum HD, Suliantari, Dewanti-Hariyadi R (2012). Multidrug resistance among different serotypes of *Salmonella* isolates from fresh products in Indonesia. Inter Food Research J.

[6] Olgunoglu IA: *Salmonella* in Fish and Fishery Products, *Salmonella* - A Dangerous Foodborne Pathogen. Dr. Barakat S M Mahmoud (Ed.), 2012. ISBN: 978-953-307-782-6, InTech, Available from: http://www.intechopen.com/books/salmonella-a-dangerous-foodborne-pathogen/salmonella-in-fish-and-fishery-products.

[7] Islam M, Morgan J, Doyle MP, Phatak SC, Millner P, Jang X (2004). Fate of *Salomella enterica* serovar Typhimurium on carrots and radishes grown in fields treated with contaminated manure composts or irrigation water. Appl Environ Microbiol.

[8] Ongeng D, Muyanja C, Geeraerd AH, Springael D, Ryckeboer J (2011). Survival of *Escherichia coli* O157:H7 and *Salmonella enterica* serovar Typhimurium in manure and manure-amended soil under tropical climatic conditions in Sub-Saharan Africa. J Appl Microbiol.

[9] Majowicz SE, Musto J, Scallan E, Angulo FJ, Kirk M, O’Brien SJ, Jones TF, Fazil A, Hoekstra RM (2010). The Global Burden of Nontyphoidal *Salmonella* Gastroenteritis. Clin Infect Dis.

[10] Dione MM, Ikumapayi UN, Saha D, Mohammed NI, Geerts S, Leven N, Adegbola RA, Antonio M (2011). Clonal differences between non-typhoidal *Salmonella* (NTS) recovered from children and animals living in close contact in the Gambia. PloS Negl Trop Dis.

[11] Reddy EA, Shaw AV, Crump JA (2010). Community-acquired bloodstream infections in Africa: a systemic review and meta-analysis. Lancet Infect Dis.

[12] Dione MM, Ikumapayi N, Saha D, Mohammed NI, Adegbola RA, Geerts S, Leven M, Antonio M (2011). Antimirobial Resistance and virulence genes of non-typhoidal *Salmonella* isolates in the Gambia and Senegal. J Infect Dev Ctries.

[13] Feasy NA, Dougan G, Kingsley RA, Heyderman RS, Gordon M (2012). Invasive non-typhoidal *salmonella* disease: an emerging and neglected tropical disease in Africa. Lancet.

[14] Bonkoungou IJO, Haukka K, Österblad M, Hakanen A, Traoré AS, Barro N, et al. Bacterial and viral etiology of childhood diarrhea in Ouagadougou, Burkina Faso. BMC Pediatr. 2013;13. 10.1186/1471-2431-13-36 PMC3616825 23506294

[15] Kagambèga A, Haukka K, Siitonen A, Traoré AS, Barro N (2011). Prevalence of *Salmonella enterica* and the hygienic indicator *Escherichia coli i*n raw meat at markets in Ouagadougou, Burkina Faso. J Food Protect.

[16] Kagambèga A, Barro N, Traoré AS, Siitonen A, Haukka K (2012). Characterization of *Salmonella enterica* and detection of the virulence genes specific to diarrheagenic *Escherichia coli* from poultry carcasses in Ouagadougou, Burkina Faso. Foodborne pathog dis.

[17] Kagambèga A, Lienemann T, Aulu L, Traoré AS, Barro N, Siitonen A, Haukka K (2013). Prevalence and characterization of *Salmonella enterica* from the feces of cattle, poultry, swine and hedgehogs in Burkina Faso and their comparison to human Salmonella isolates. BMC Microbiol.

[18] Katukiza AY, Ronteltap M, van der Steen P, Foppen JWA, Lens PNL (2013). Quantification of microbial risks to human health caused by waterborne viruses and bacteria in an urban slum. J Appl Microbiol.

[19] Akinyemi KO, Iwalokun BA, Foli F, Oshodi K, Coker AO (2011). Prevalence of multiple drug resistance and screening of enterotoxin (stn) gene in *Salmonella enterica* serovars from water sources in Lagos, Nigeria. Public Health.

[20] Galanis E, Danilo MA, Wong LF, Patrick ME, Binsztein N, Cieslik A, Chalermchaikit T, Aidara-Kane A, Ellis A, Angulo FJ, Wegener HC (2006). Web-based Surveillance and Global *Salmonella* Distribution, 2000–2002. Emerg Infect Dis.

[21] Dembélé R, Konaté A, Bonkoungou IJO, Kagambèga A, Konaté K, Bagré TS, Traoré AS, Barro N (2014). Serotyping and antimicrobial susceptibility of *Salmonella* isolated from children under five years of age with diarrhea in rural Burkina Faso. Afr J Microbiol Res.

[22] Ateba BH, Nougang ME, Noah EO, Tamatcho KB, Tawedi RE, Mbilongo J, Nola M, Njine T (2012). Occurrence of *Salmonella* spp in surface waters of Yaoundé, Cameroon. J Environ Sci Water Resource.

[23] Catalao Dionisio LP, Joao M, Ferreiro VS, Fidalgo ML, García Rosado ME, Borrego JJ (2000). Occurrence of *Salmonella spp*. in estuarine and coastal waters of Portugal. Antonie van Leeuwenhoek.

[24] Martinez-Urtaza J, Saco M, de Novoa J, Perez-Pineiro P, Peiteado J, Lozano-Leon A, Garcia-Martin O (2004). Influence of environmental factors and human activity on the presence of *Salmonella* serovars in a marine environment. Appl Environ Microbiol.

[25] Haley BJ, Cole DJ, Lipp EK (2009). Distribution, diversity, and seasonality of waterborne Salmonellae in a rural watershed. Appl Environ Microbiol.

[26] Gorski L, Parker CT, Liang A, Cooley MB, Jay-Russell MT, Gordus AG, Atwill ER, Mandrell RE (2011). Prevalence, distribution, and diversity of *Salmonella enterica* in a major produce region of California. Appl Environ Microbiol.

[27] Martinez-Urtaza J, Liebana E (2005). Use of pulsed-field gel electrophoresis to characterize the genetic diversity and clonal persistence of *Salmonella* senftenberg in mussel processing facilities. Int J Food Microbiol.

[28] Awuor WS, Miruka OD, Eliud WN (2011). Characterisation of *Salmonella* Isolated from Nile Tilapia (Oreochromis niloticus) along Lake Victoria Beaches in Western Kenya. Inter J Biol Med Sciences.

[29] Onyango MD, Wandili S, Kakai R, Waindi ED (2009). Isolation of *Salmonella* and Shigella from fish harvested from the Winam Gulf of Lake Victoria, Kenya. J Infect Dev Ctries.

[30] Hammack TS, Jacobson AP, Andrews WH (2008). The effect of preenrichment and selective enrichment media on recovery of Salmonella Typhi from the tropical fruit mamey. J AOAC Int.

[31] Islam M, Morgan J, Doyle MP, Phatak SC, Millner P, Jang X (2004). Persistence of *Salmonella enterica* serovar Typhimurium on lettuce and parsley and in soils on which they were grown in fields treated with contaminated water. Foodborne Pathog Dis.

[32] Lienemann T, Niskanen T, Guedes S, Siitonen A, Kuusi M, Rimhanen-Finne R (2011). Iceberg lettuce as suggested source of a nationwide outbreak caused by two *Salmonella* serotypes, Newport and Reading, in Finland in 2008. J Food Prot.

[33] Traoré O, Martikainen O, Siitonen A, Traoré AS, Barro N, Haukka K (2014). Occurrence of *Vibrio cholerae* in fish and water from a reservoir and a neighbouring channel in Ouagadougou/ Burkina Faso. J Infect Dev Ctries.

[34] Kauffmann F (1971). Classification and nomenclature of the genus *Salmonella*. Acta Pathol Microbiol Scand [B] Microbiol Immunol.

